# Topical and oral peroxisome proliferator-activated receptor-α agonist ameliorates diabetic corneal neuropathy

**DOI:** 10.1038/s41598-024-64451-4

**Published:** 2024-06-11

**Authors:** Hassan Mansoor, Isabelle Xin Yu Lee, Molly Tzu-Yu Lin, Heng Pei Ang, Yao Cong Xue, L. Krishaa, Moushmi Patil, Siew-Kwan Koh, Hong Chang Tan, Lei Zhou, Yu-Chi Liu

**Affiliations:** 1 Al Shifa Trust Eye Hospital, Rawalpindi, Pakistan; 2 Tissue Engineering and Stem Cell Group, Singapore Eye Research Institute, 11 Third Hospital Ave, Singapore, 168751 Singapore; 3 Ocular Proteomic Group, Singapore Eye Research Institute, Singapore, Singapore; 4 Department of Endocrinology, Singapore General Hospital, Singapore, Singapore; 5 Department of Applied Biology and Chemical Technology, School of Optometry, Research Centre for SHARP Vision (RCSV), The Hong Kong Polytechnic University, Hung Hom, Hong Kong; 6 Centre for Eye and Vision Research (CEVR), 17W Hong Kong Science Park, Pak Shek Kok, Hong Kong; 7 Cornea and Refractive Surgery Group, Singapore Eye Research Institute, Singapore, Singapore; 8 Department of Cornea and External Eye Disease, Singapore National Eye Centre, Singapore, Singapore; 9 Eye-Academic Clinical Program, Singapore Graduate Medical School, Duke-National University, Singapore, Singapore; 10 Department of Ophthalmology, National Taiwan University Hospital, Taipei, Taiwan

**Keywords:** Translational research, Corneal diseases

## Abstract

Diabetic corneal neuropathy (DCN) is a common diabetic ocular complication with limited treatment options. In this study, we investigated the effects of topical and oral fenofibrate, a peroxisome proliferator-activated receptor-α agonist, on the amelioration of DCN using diabetic mice (n = 120). Ocular surface assessments, corneal nerve and cell imaging analysis, tear proteomics and its associated biological pathways, immuno-histochemistry and western blot on PPARα expression, were studied before and 12 weeks after treatment. At 12 weeks, PPARα expression markedly restored after topical and oral fenofibrate. Topical fenofibrate significantly improved corneal nerve fibre density (CNFD) and tortuosity coefficient. Likewise, oral fenofibrate significantly improved CNFD. Both topical and oral forms significantly improved corneal sensitivity. Additionally, topical and oral fenofibrate significantly alleviated diabetic keratopathy, with fenofibrate eye drops demonstrating earlier therapeutic effects. Both topical and oral fenofibrate significantly increased corneal β-III tubulin expression. Topical fenofibrate reduced neuroinflammation by significantly increasing the levels of nerve growth factor and substance P. It also significantly increased β-III-tubulin and reduced CDC42 mRNA expression in trigeminal ganglions. Proteomic analysis showed that neurotrophin signalling and anti-inflammation reactions were significantly up-regulated after fenofibrate treatment, whether applied topically or orally. This study concluded that both topical and oral fenofibrate ameliorate DCN, while topical fenofibrate significantly reduces neuroinflammation.

## Introduction

The cornea is the most densely innervated tissue in the human body. Corneal innervation maintains ocular surface integrity and ocular surface homeostasis by releasing neuromediators, mediating reflex tears production and blinking^1,2^. Metabolic changes when diabetes mellitus (DM) is not controlled cause corneal nerves to degenerate and apoptose, resulting in diabetic corneal neuropathy (DCN)^1,3^. DCN affects 47–64% of DM patients and is characterized by corneal hypoesthesia, reduced blink rate, decreased reflex tear secretion, and tear film instability. These pathological alterations contribute to ocular surface breakdown, neurotrophic ulceration, and even sight-threatening corneal infection or perforation^1^. Current management of DCN is mainly symptomatic treatment and does not target DCN pathogenesis. Topical recombinant human nerve growth factor (rhNGF) is the only commercially available treatment that has shown a beneficial effect on corneal nerve regeneration and was found to improve corneal subbasal nerve density in patients with neurotrophic keratopathy after 8 weeks of treatment^4^. However, the high cost and the need for frequent applications of rhNGF have limited its widespread adoption. Hence clinicians and researchers are striving to identify new treatment strategies for DCN to promote corneal nerve regeneration or prevent nerve degeneration.

Peroxisome proliferator-activated receptors (PPARs) have three isoforms, identified as PPARα, PPARβ/δ, and PPARγ, and corneas mainly present PPARα^5,6^. Fenofibrate, a PPARα agonist, modulates lipid metabolism and is conventionally used to treat hyperlipidemia^7^. It is believed that deranged lipid metabolism participates in the development and progression of diabetic peripheral neuropathy (DPN) by triggering neural vascular insufficiency, deteriorating mitochondrial function due to oxidative stress, and impairing the conduction of electrical impulses in the neurons^8–10^. Therefore, normalising lipid metabolism could mitigate the risk of developing DPN^9^. Oral administration of fenofibrate prevents the development of sciatic neuropathy in diabetic mice by inhibiting neural and endothelial damage via activation of the PPARα-AMPK-PGC-1α-eNOS-NO pathway^11^. The fenofibrate intervention and event lowering in diabetes (FIELD) study also reported the neuroprotective effect of the PPARα agonist as its systemic use reduced the overall neuropathy and improved the reversal of pre-existing neuropathy in type 2 diabetics, although the mechanism remained unknown^12^.

More recently, the protective effect of oral fenofibrate against corneal neurodegeneration has been reported in both diabetic rats and humans. Ma et al. demonstrated that 2-months of oral fenofibrate treatment significantly altered corneal neurotrophic factors, including NGF and glial cell-derived neurotrophic factor, and subsequently improved the corneal nerve fibre density (CNFD) and corneal epitheliopathy in diabetic rats, implying the potential beneficial effects of fenofibrate in DCN^6^. Likewise, we found that oral fenofibrate prevented corneal nerve degeneration and stimulated corneal nerve regeneration in patients with type II DM^13^. However, our study could not uncover the tissue responses and underlying mechanisms on a cellular level. Such clarification would require laboratory analysis of corneas from subjects treated with fenofibrate. Furthermore, we also believe that the administration of fenofibrate as eye drops will reach the corneal nerve plexus faster and at higher concentrations, and fenofibrate eye drops will have similar or even greater efficacy than oral fenofibrate. Fenofibrate eye drops would also pave the way for better clinical adoption and patient compliance in ophthalmology clinics.

In the present study, we used a diabetic mice model to compare the ability of topical fenofibrate to oral fenofibrate in stimulating corneal nerve regeneration. We conducted comprehensive analyses, ranging from clinical ocular surface functional assessment, corneal imaging data, and tissue responses to molecular analysis, including proteomic profiles, to elucidate the efficacy and mechanisms underlying the potential neuroprotective effects of topical and oral fenofibrate.

## Results

### Blood glucose level

The negative control (NC) group consisted of 60 eyes from 30 wild-type mice, whereas the positive control (PC), topical fenofibrate (TF), and oral fenofibrate (OF) groups consisted of 180 eyes from 90 Akita mice, with each group consisting of 60 eyes. Diabetic mice, including PC, TF and OF groups, presented significantly higher blood glucose levels than the NC group, at all time points (all p < 0.001; Supplementary Fig. 1). There was no significant difference in the blood glucose level between the PC, TF and OF groups throughout the study period.

### PPARα expression on diabetic corneas was restored after topical and oral fenofibrate treatment

**Figure 1:**
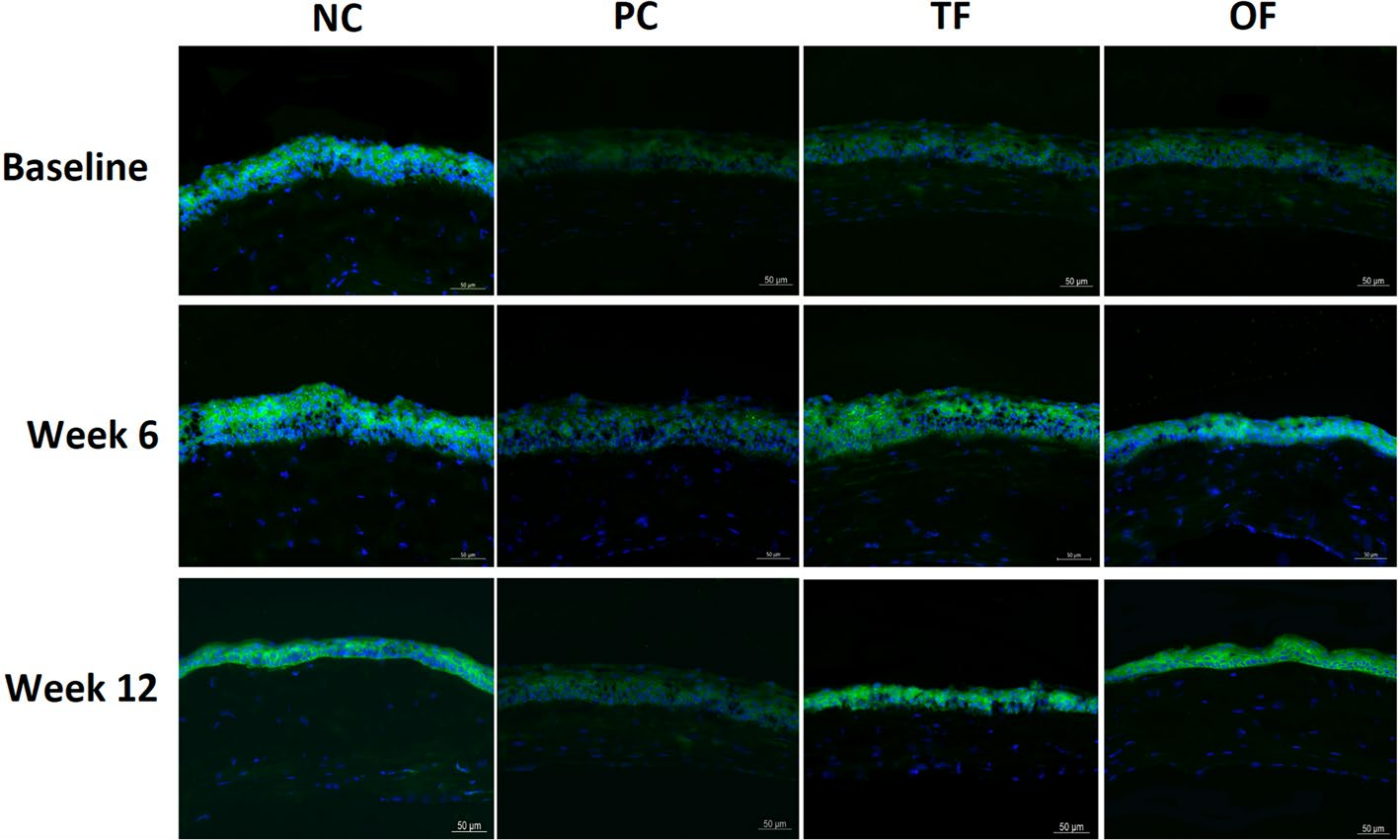
Representative corneal section images of mice of different experimental groups showing PPARα immunostaining. Intense PPARα immunosignals were observed in the corneal epithelial layer of non-diabetic mice (NC group) compared to diabetic mice (PC, TF and OF groups). PPARα expression increased after topical and oral fenofibrate treatment in diabetic corneas. Scale bar: 50 μm.

In non-diabetic corneas, intense PPARα signals were observed throughout the corneal epithelial layer. PPARα expression was markedly downregulated in the diabetic corneas, including PC, TF and OF groups. After treatment, PPARα expression was noticeably increased in the TF and OF groups at 6 weeks and 12 weeks, compared to the PC group (Fig. 1).

### Diabetic corneas presented with thicker corneal thickness

**Figure 2:**
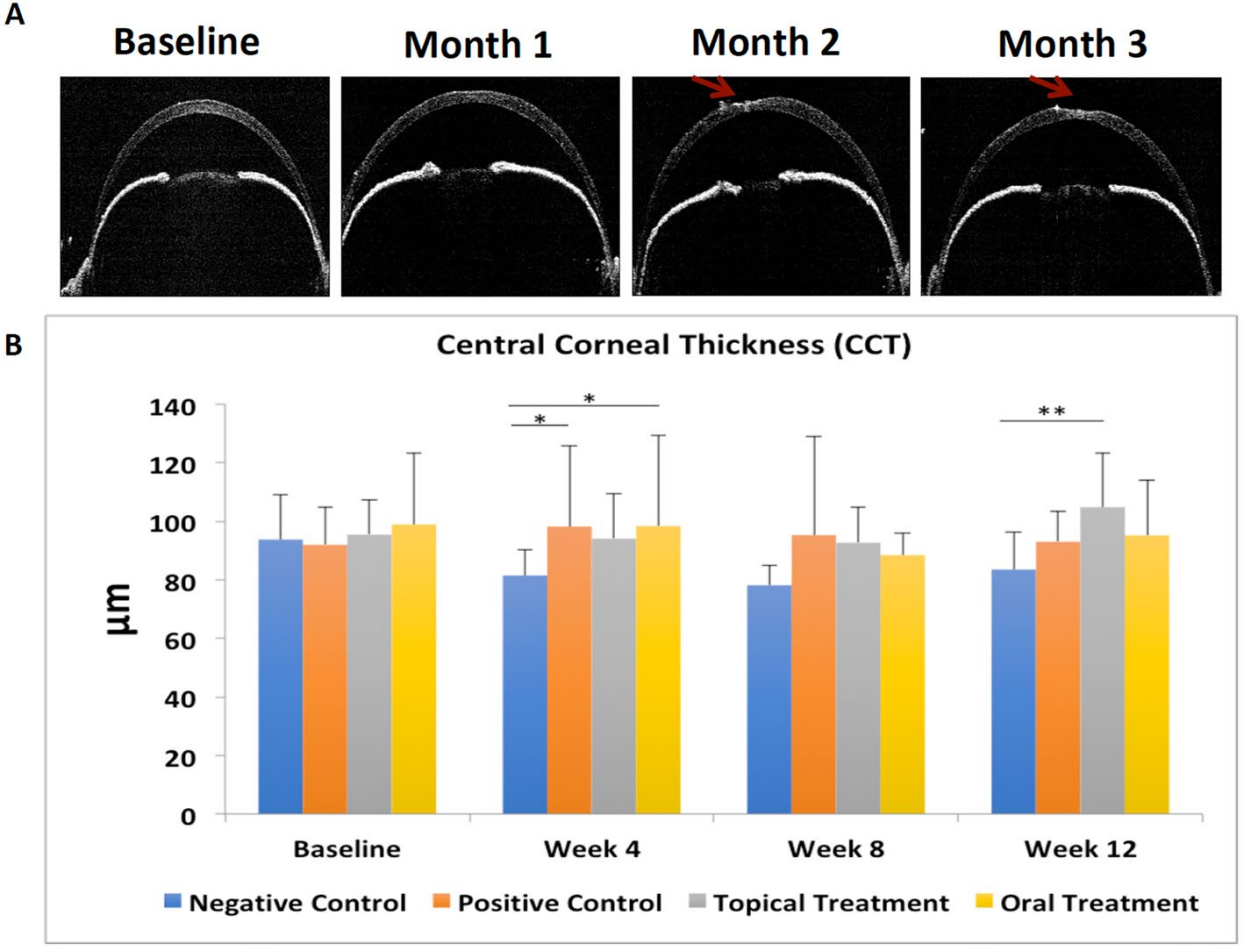
Effects of fenofibrate treatment on CCT for 4 groups at different time points. **(A)** Mice in the PC group tended to develop neurotrophic keratitis, which manifested as impaired corneal healing and focal corneal ulceration (red arrow). (**B)** Diabetic mice presented an increased CCT compared to non-diabetic mice at 4 weeks. Asterisk represents p < 0.05, double asterisk represents p < 0.01.

Diabetic mice in the PC, TF and OF groups presented an increased CCT compared to the NC group, at 4 weeks (PC versus NC groups: p = 0.03; OF versus NC groups: p = 0.03), and at 12 weeks (TF versus NC groups: p = 0.004). Additionally, mice in the PC group tended to develop neurotrophic keratopathy, presenting with impaired corneal healing and focal corneal ulceration (Fig. 2A). No significant difference was observed between the CCT of the PC, TF and OF groups at different time points (Fig. 2B).

### Topical and oral fenofibrate significantly improved the corneal nerve metrics in diabetic corneas

**Figure 3:**
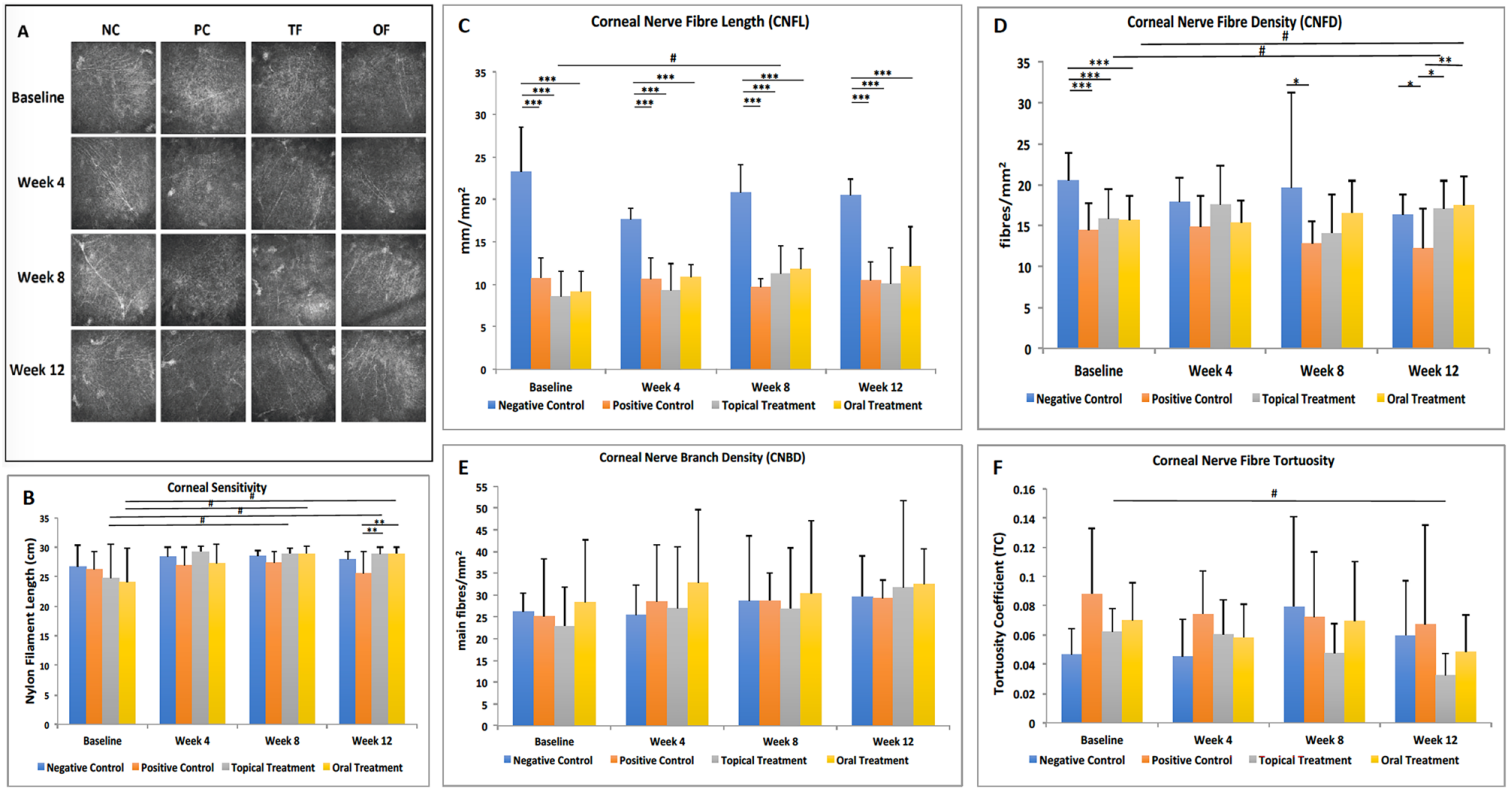
Effects of fenofibrate treatment on corneal sensitivity and corneal nerve metrics for 4 groups at different time points. **(A)** Representative IVCM images of corneal nerves of 4 groups at different time points. The bar charts showing the **(B)** corneal sensitivity of 4 groups at different time points. The bar charts showing the **(C)** CNFL, **(D)** CNFD, **(E)** CNBD and **(F)** tortuosity coefficient of 4 groups at different time points. Asterisk and hash represent p < 0.05, double asterisk represents p < 0.01, triple asterisk represents p < 0.001. Asterisk, double asterisk and triple asterisk represent statistical significance for intergroup comparisons. Hash indicates statistical significance for comparisons across different time points.

At baseline, diabetic mice in the PC, TF and OF groups had significantly reduced CNFL and CNFD in comparison to the NC group (all p < 0.001) (Fig. 3C, D). After topical treatment, CNFL significantly improved at 8 weeks (p = 0.04). A significant improvement was also observed in the CNFD (p = 0.01) and TC (p = 0.01) in the TF group at 12 weeks (Fig. 3C,D,F). Likewise, oral fenofibrate significantly improved the CNFD at 12 weeks (p = 0.01) (Fig. 3D). In both TF and OF groups, CNFD improved to a level comparable with that of the NC group at 12 weeks (Fig. 3D). Moreover, there was a significant difference in the CNFD between the PC and TF groups (p = 0.01), as well as between the PC and OF groups (p = 0.008), highlighting the stimulating effect of topical and oral fenofibrate treatment on diabetic corneal nerve degeneration (Fig. 3D).

### Corneal sensitivity significantly improved after topical and oral fenofibrate treatment

The corneal sensitivity improved significantly in the TF group at 8 and 12 weeks compared to that at baseline (p = 0.04 and p = 0.04, respectively). The corneal sensitivity also recovered in the OF group at 8 and 12 weeks in comparison to that at baseline (p = 0.04 and p = 0.04, respectively). At 12 weeks, mice with topical and oral fenofibrate treatment presented significantly better corneal sensitivity than those in the PC group (p = 0.004 and p = 0.004, respectively) (Fig. 3B). These significant differences could be attributed to the continued reduction of the corneal sensitivity in the PC group (Fig. 3B).

### Topical and oral fenofibrate effectively increased corneal epithelial cell density and ameliorated diabetic keratopathy

**Figure 4:**
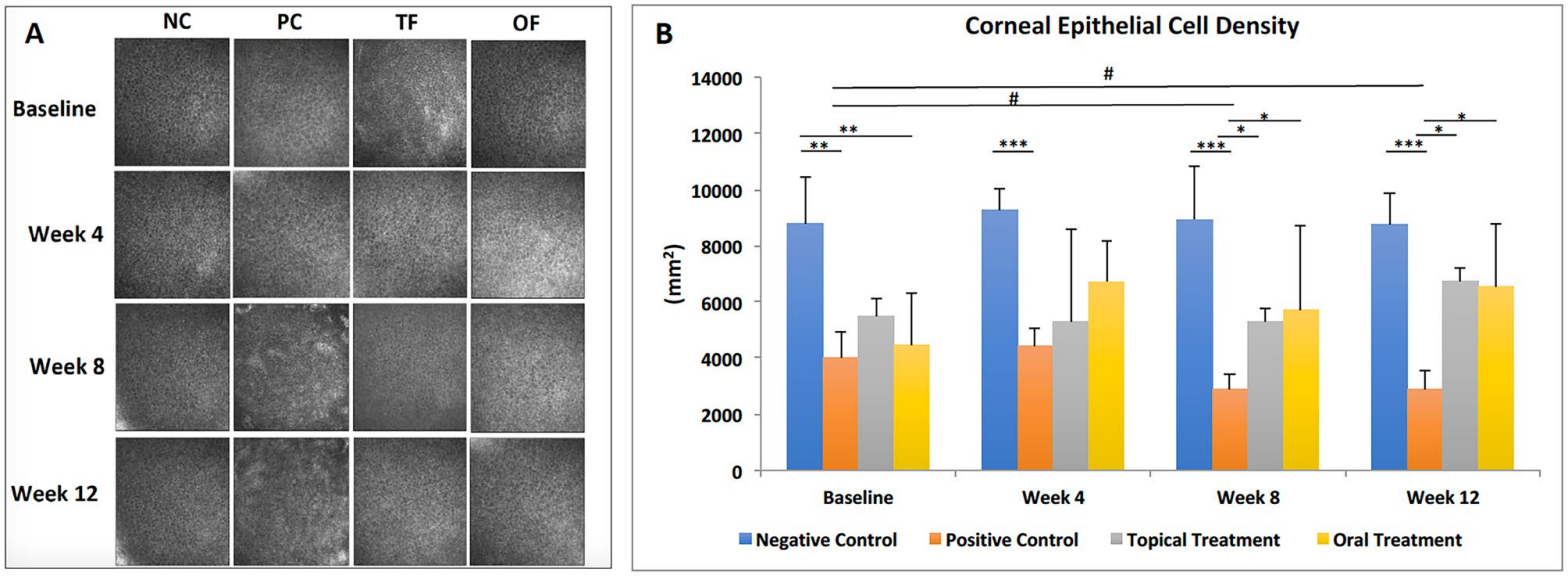
Corneal epithelial morphology and CECD for 4 groups at different time points. **(A)** The morphology of corneal epithelial cells shows restoration of regular cellular polygonal shape, decreased cell size, and enhanced CECD in TF and OF group compared to PC. (**B)** The bar chart showing that fenofibrate treatment increased CECD in TF and OF groups compared to PC group at 8 weeks and 12 weeks. Asterisk and hash represent p < 0.05, double asterisk represents p < 0.01, triple asterisk represents p < 0.001. Asterisk, double asterisk and triple asterisk represent statistical significance for intergroup comparisons. # indicates statistical significance for comparisons across different time points.

On IVCM images, diabetic mice at baseline presented with increased variability in corneal epithelial cell size, wider intracellular space, more haze, and reduced corneal epithelial cell density (CECD) compared to the non-diabetic mice (Fig. 4A). In this study, cell density was found to be lowered in diabetic mice than NC group, especially in PC (p = 0.003) and OF (p = 0.007) groups, at baseline. During the study period, the CECD in the PC group continued to decrease, hence a significant reduction was noted at 8 weeks (p = 0.01) and 12 weeks (p = 0.01) compared to the baseline. Consequently, a significant difference between the CECD of PC and NC groups was observed at 4, 8 and 12 weeks (all p < 0.001). After treatment, TF and OF groups presented significantly better CECD than that in the PC group, at 8 and 12 weeks (all p < 0.05) (Fig. 4B).

**Figure 5:**
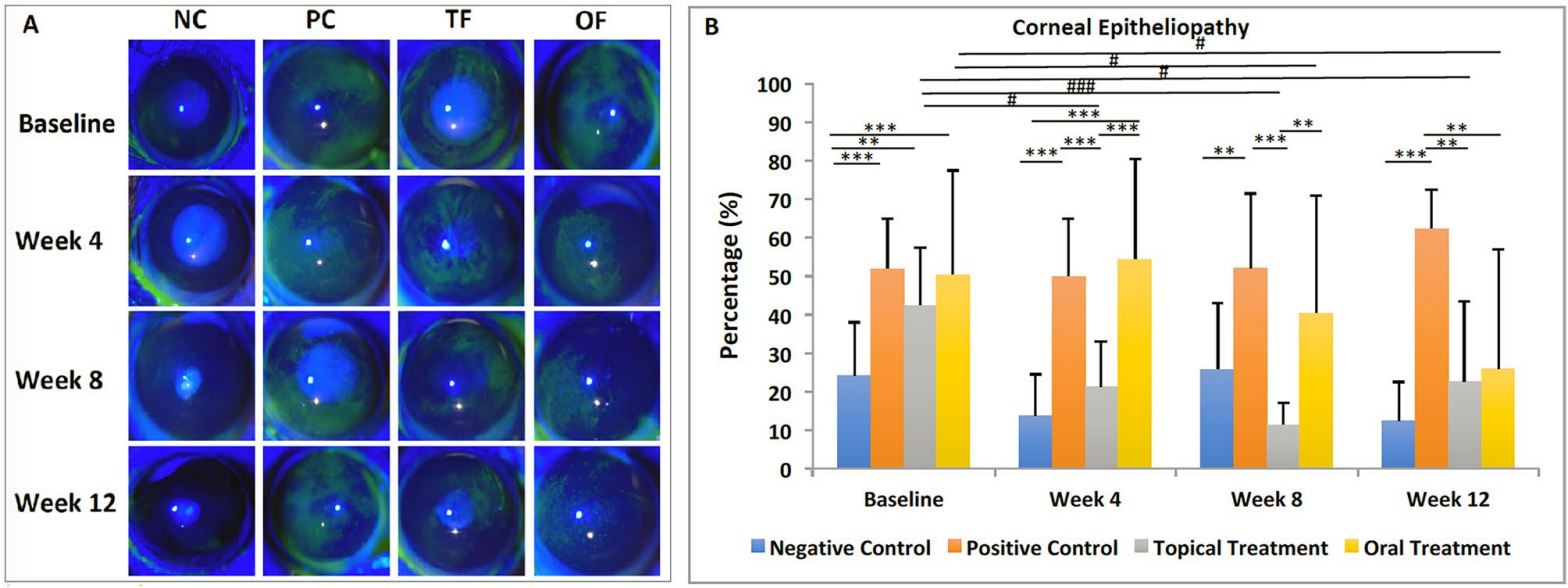
Slit lamp evalution of diabetic keratopathy for 4 groups at different time points. **(A)** Representative slit-lamp images and **(B)** bar chart showing that topical and oral fenofibrate treatment significantly improved diabetic keratopathy in diabetic mice. Fenofibrate eye drops were more efficacious than oral treatment in reducing corneal epitheliopathy at 4 weeks and 8 weeks. Hash represents p < 0.05, double asterisk represents p < 0.01, triple asterisk and triple hash represent p < 0.001. Hash and triple hash indicate statistical significance for comparisons across different time points. Double asterisk and triple asterisk represent statistical significance for intergroup comparisons.

On slit-lamp evaluation, diabetic mice, including PC, TF and OF groups, presented with a significantly worse diabetic epitheliopathy, evidenced by a larger corneal positive-staining area at baseline (all p < 0.05 when compared with NC, Fig. 5A, B). After treatment, corneal epitheliopathy significantly improved in the TF group at 4 weeks (p = 0.01), 8 weeks (p < 0.001) and 12 weeks (p = 0.01), in comparison with that of baseline. Despite significant improvement, some impaired corneal healing was visible in the TF group at week 12, although no focal ulceration was found. An improvement in epitheliopathy was also observed in the OF group at 8 weeks (p = 0.04) and 12 weeks (p = 0.02). Notably, our study results revealed that oral fenofibrate required 12 weeks for a significant response in alleviating diabetic keratopathy, whereas topical fenofibrate demonstrated a significant response after 4 weeks.

### Topical and oral fenofibrate promoted corneal nerves regeneration in diabetic corneas

**Figure 6:**
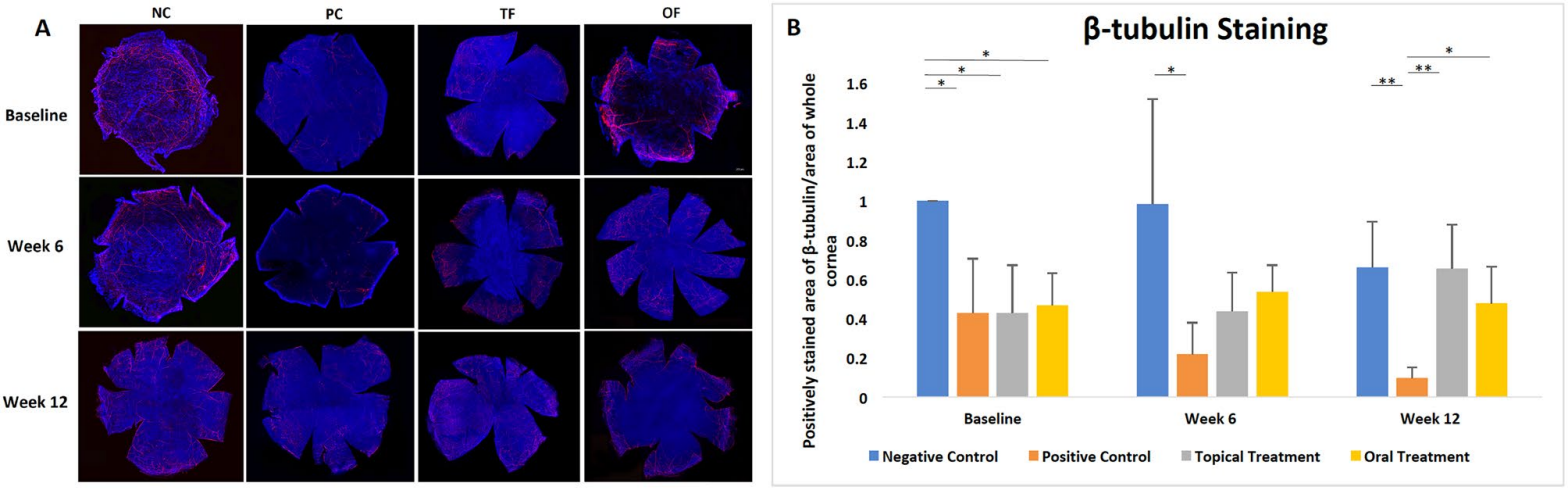
β-tubulin staining and quantification of the whole mount corneal sections of 4 groups at different time points. (**A**) Whole mount corneal sections and (**B**) bar chart showing that intensity of immunosignals and density of positively stained nerves were significantly lower in diabetic mice (PC, TF and OF groups) compared to non-diabetic mice (NC group), however they increased after both topical and oral fenofibrate treatment. Asterisk represents p < 0.05, double asterisk represents p < 0.01. Asterisk and double asterisk represent statistical significance for intergroup comparisons.

The density of nerve trunks and branches immunostained with β-III tubulin was higher in the periphery than in the central cornea. At baseline, the immunosignals from positively-stained nerves were noticeably reduced in the diabetic mice (PC, TF and OF groups) compared to the non-diabetic mice (NC group) (all p = 0.04) (Fig. 6A, B). After 12 weeks treatment, the intensity of immunosignals and positively-stained nerves was significantly higher in the TF and OF groups compared to the PC group (p = 0.009 and p = 0.03, respectively) (Fig. 6B).

### Topical fenofibrate treatment significantly suppressed the neuroinflammation on diabetic ocular surface

**Figure 7:**
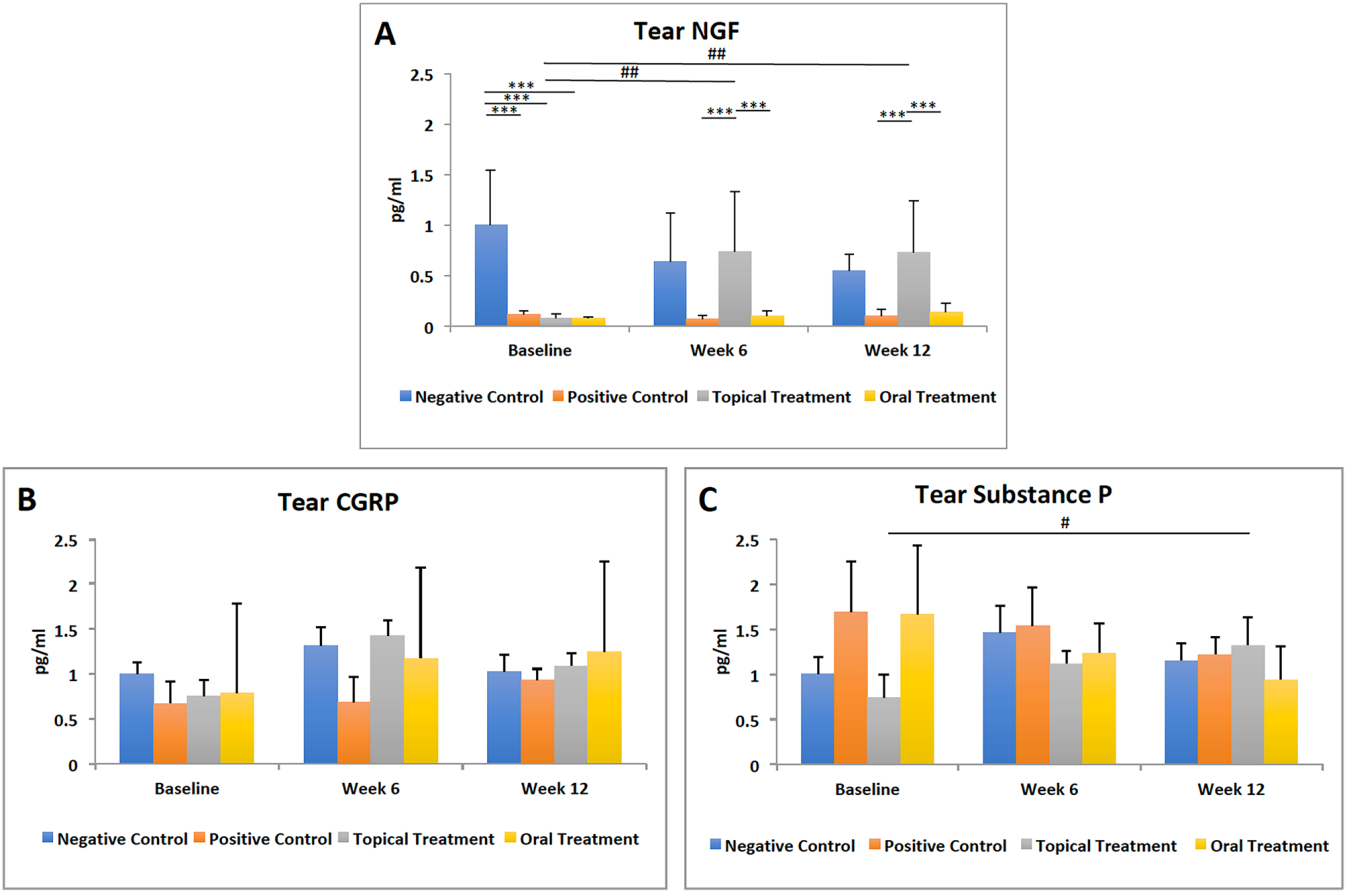
Tear neuromediators profile for 4 groups at different time points. In contrast to oral treatment, topical fenofibrate treatment increased **(A)** tear NGF levels at 6 weeks and 12 weeks as well as **(C)** substance P levels at 12 weeks compared to baseline. **(B)** Fenofibrate treatment did not significantly alter the tear CGRP levels. Hash represents p < 0.05, double hash represents p < 0.01, triple asterisk represents p < 0.001. Hash and double hash indicate statistical significance for comparisons across different time points. Triple asterisk represents statistical significance for intergroup comparisons.

The tear NGF level at baseline was significantly reduced in the diabetic mice compared to the non-diabetic mice (all p < 0.001). After treatment, tear NGF levels significantly increased in the TF group at 6 weeks (p = 0.006) and 12 weeks (p = 0.006), achieving levels comparable to those in the NC group. Consequently, a significant difference was evident at 6 weeks and 12 weeks between the TF and PC groups (p < 0.001 and p < 0.001, respectively) and TF and OF groups (p < 0.001 and p < 0.001, respectively). Fenofibrate eye drops also increased the tear SP levels at 12 weeks compared to baseline (p = 0.03). Oral fenofibrate treatment did not significantly alter the tear NGF and SP levels (Fig. 7A–C).

### Topical and oral fenofibrate downregulated CDC42 mRNA expression in trigeminal ganglions

**Figure 8:**
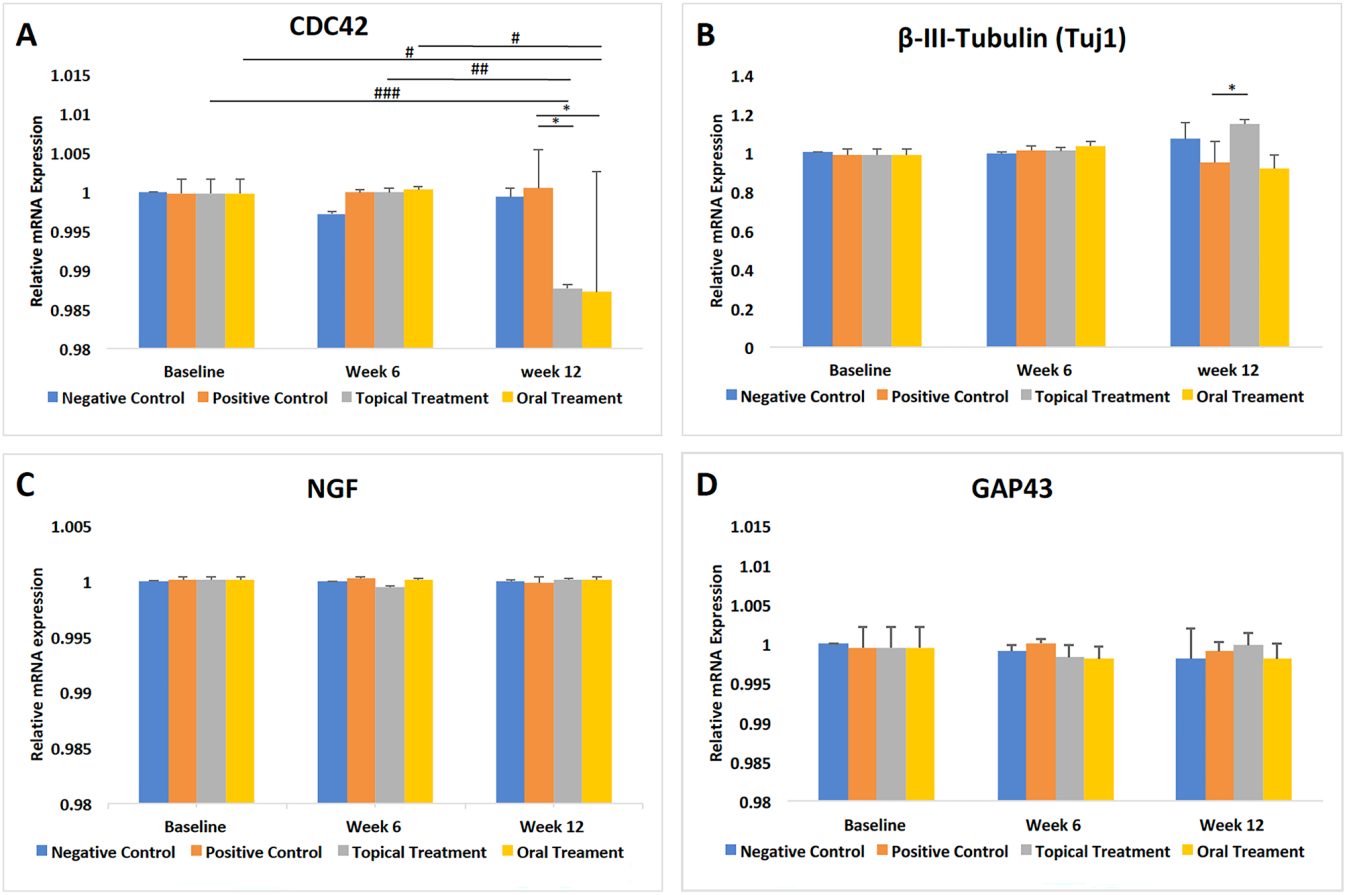
mRNA expression in trigeminal ganglion for 4 groups at different time points. **(A)** Topical and oral fenofibrate treatment significantly decreased CDC42 mRNA expression at 12 weeks compared to baseline. **(B)** Topical fenofibrate treatment significantly increased β-III-tubulin (Tuj1) mRNA expression at 12 weeks compared to PC group. No significant difference was observed in **(C)** NGF and **(D)** GAP43 mRNA expression between groups at different time points. Asterisk and hash represent p < 0.05, double hash represents p < 0.01, triple hash represents p < 0.001. Asterisk represents statistical significance for intergroup comparisons. Hash, double hash and triple hash indicate statistical significance for comparisons across different time points.

In both TF and OF groups, the relative CDC42 mRNA expression was significantly downregulated at 12 weeks (p < 0.001 and p = 0.02, repsectively). A time-point comparison between 6 versus 12 weeks in both TF and OF groups revealed a significant reduction of the CDC42 mRNA expression (p = 0.002 and p = 0.01, respectively). At 12 weeks, fenofibrate-treated diabetic mice in the TF and OF groups presented markedly reduced CDC42 mRNA expression compared to the PC group (p = 0.01 and p = 0.02, respectively, (Fig. 8A). An enhanced β-III-Tubulin (Tuj1) mRNA expression was also observed in the TF group in comparison to the PC group (p = 0.03) at 12 weeks (Fig. 8B).

### PPARα protein expression improved after topical fenofibrate treatment

**Figure 9:**
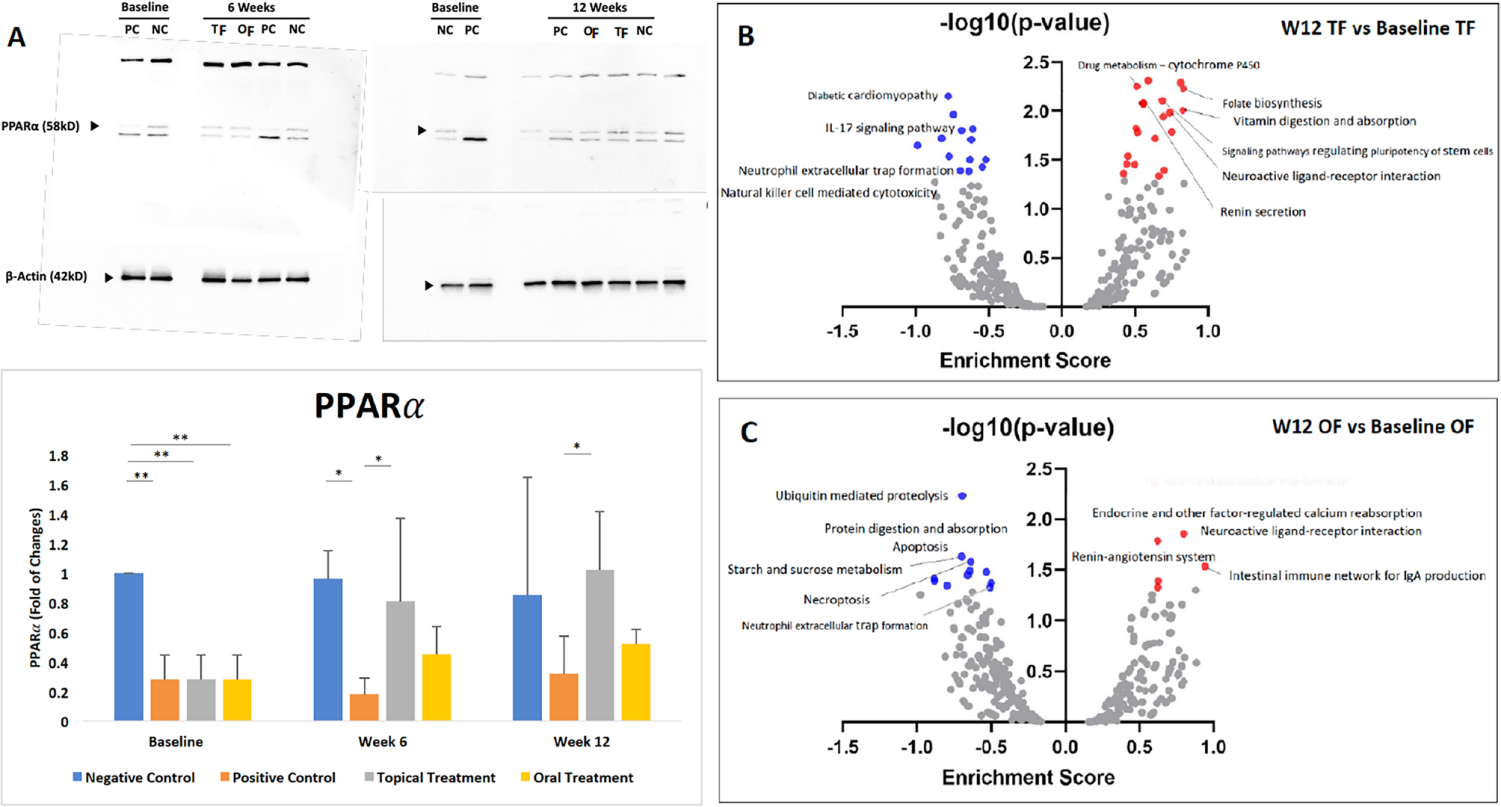
PPARα protein expression and GSEA plots. **(A)** PPARα protein expression analysis for 4 groups at different time points show that the expression was significantly lower in the corneas of diabetic mice (PC, TF, and OF groups) compared to non-diabetic mice (NC group) at baseline, but it increased with both topical and oral fenofibrate treatment. Asterisk represents p < 0.05, double asterisk represents p < 0.01. Scatter plots for GSEA results [ES and −log10 (p value)] showing the enrichment pathways (p < 0.05) at **(B)** 12 weeks versus baseline in TF group, and **(C)** 12 weeks versus baseline in OF group. Red dots represent the significantly up-regulated pathways, and blue dots represent significantly down-regulated pathway.

Although the PPARα protein expression was significantly reduced in the diabetic corneas (PC, TF and OF groups) compared to non-diabetic corneas (all p = 0.009) at baseline, it notably increased with fenofibrate treatment (Fig. 9A). A significant difference was observed between the PC and NC groups (p = 0.03), and the PC and TF groups (p = 0.04) at 6 weeks, as well as between the PC and TF groups (p = 0.04) at 12 weeks. In the TF and OF groups, corneal PPARα protein expression increased by 3.64-folds and 1.86-folds, respectively, at 12 weeks (Fig. 9A).

### Topical and oral fenofibrate treatment up-regulated the neurotrophin signalling pathway and suppressed neutrophil reactions in diabetic corneas

Diabetic mice presented with significantly reduced complement and coagulation cascade activity (ES = −0.77; p = 0.002), significantly reduced cholesterol metabolism (ES = −0.76; p = 0.004), as well as significantly increased adipocytokine signalling pathway (ES = 0.97, p = 0.006) (Supplementary Fig. 2A and Supplementary Table 3). After 12 weeks of fenofibrate treatment, neuroactive ligand-receptor interaction significantly increased in both the TF (ES = 0.82; p = 0.02) and OF groups (ES = 0.79; p = 0.01), as did signalling pathways regulating the pluripotency of stem cells in the TF group (ES = 0.75; p = 0.02), while neutrophil extracellular trap formation was significantly reduced in both the TF (ES = −0.61; p = 0.01) and OF groups (ES = −0.60; p = 0.004; Fig. 9B, C and Supplementary Tables 4 and 5). Likewise, necroptosis (ES = −0.51; p = 0.04) and apoptosis were significantly reduced (ES = -0.88; p = 0.04) in the OF group. When comparing the TF with OF treatment, sphingolipid metabolism was noted to be significantly higher in the former (ES = 0.86; p = 0.008) (Supplementary Fig. 2B and Supplementary Table 6). Table 1 summarizes the results and differences between the topical fenofibrate and oral fenofibrate in DCN treatment.

**Table 1:** Summary of comparison of topical fenofibrate and oral fenofibrate in DCN treatment.

Parameters	Comparison of topical fenofibrate and oral fenofibrate in DCN treatment
PPARα Immunostaining	PPARα immunostaining in the corneal epithelium was noticeably increased in the TF and OF groups at 6 weeks and 12 weeks, compared to the PC group
Central corneal thickness	No significant difference between the TF and OF groups at different time points
Corneal nerve metrics	TF improved CNFL at 8 weeks, and CNFD and TC at 12 weeks. OF only improved CNFD at 12 weeks
Corneal sensitivity	Both TF and OF groups presented with significantly better corneal sensitivity at week 12
Corneal epithelial cell density (CECD)	Both TF and OF groups had significantly improved CECD than the PC group at 8 weeks and 12 weeks
Ocular surface integrity	TF alleviated diabetic keratopathy earlier (4 weeks) than OF treatment (12 weeks)
Corneal whole mount β-tubulin staining	Both TF and OF groups presented with significantly higher intensity of staining on nerves at week 12 compared to PC group
Tear neuromediators	TF increased the tear NGF and SP levels at 12 weeks, whereas, OF did not
Trigeminal ganglion mRNA expression	TF increased β-III-Tubulin mRNA expression relative to the PC group at week 12. Both TF and OF groups presented with markedly reduced CDC42 mRNA expression at week 12 compared to the PC group
PPARα protein expression on corneas	TF and OF groups showed a 3.63-fold and 2.07-fold increase in corneal PPARα protein expression, respectively, after 12 weeks treatment
Gene set enrichment analysis (GSEA)	Following pathways increased after 12 weeks treatment: ∙ TF group: neuroactive ligand-receptor interaction, pluripotency of stem cells, Sphingolipid metabolism ∙ OF group: neuroactive ligand-receptor interaction Following pathways decreased after 12 weeks fenofibrate treatment: ∙ TF group: neutrophil extracellular trap formation ∙ OF group: neutrophil extracellular trap formation, necroptosis and apoptosis

## Discussion

While researchers investigate the pathogenic mechanisms underlying DCN, effective treatment options for DCN are also being explored. Our study results showed that fenofibrate improved CNFL, CNFD, and TC in the TF group but only CNFD in the OF group, with corneal sensitivity recovery comparable in both groups. Both topical and oral fenofibrate effectively increased CECD in diabetic corneas and ameliorated diabetic keratopathy, with fenofibrate eye drops showing earlier beneficial effects. On the molecular level, topical fenofibrate decreased CDC42 mRNA expression while increasing β-III-tubulin mRNA expression in trigeminal ganglions, and increased NGF and SP levels in tears, reducing neuroinflammation. Quantitative proteomic analyses revealed that oral and topical fenofibrate significantly up-regulated the neuroactive ligand-receptor interaction and reduced neutrophil formation.

While oral fenofibrate demonstrated positive treatment efficacy on DCN, topical formulation would significantly increase clinical use, adoption in ophthalmic clinics and patient compliance. Moreover, topical fenofibrate would be a preferred choice as the drug concentration in the corneas would be higher than after systemic fenofibrate administration. It also eliminates the systemic side effects associated with oral fenofibrate intake. Furthermore, topical fenofibrate can be explored as an adjuvant treatment to improve the nerve status in DM patients undergoing corneal surgery that impair corneal nerves.

PPARα expression has been demonstrated in the mouse, rat, and human corneas, with reportedly high levels in the epithelium^6^. Matlock et al. discovered that diabetes markedly decreased the PPARα expression not only in the human cornea but also in the pancreas and retina^6,15, 16^. In our study, PPARα immunochemistry expression and protein levels in the corneal epithelium were significantly lower in diabetic mice, but they increased with topical and oral fenofibrate treatment, with greater PPARα expression observed in the TF group. Furthermore, fenofibrate treatment stimulated and restored the corneal nerve metrics, as evidenced by significantly improved CNFL, CNFD, and TC in the TF group, as well as CNFD in the OF group, through the activation of the PPARα pathway and upregulation of the neuroactive ligand-receptor interaction that were identified in the GSEA. As a result, the corneal sensitivity in both the TF and OF groups improved significantly, and mice treated with topical or oral fenofibrate had significantly better corneal sensitivity than those in the PC group. Matlock and colleagues also reported a protective effect of oral fenofibrate on diabetes-induced deterioration in corneal nerve metrics in streptozotocin-induced diabetic rats and mice, which supports our findings^6^.

Tear neuromediators, such as SP and NGF, play an important role in maintaining corneal neuronal health, promoting neuronal regeneration and wound healing in DM^1,17, 18^. Tear SP has been shown to modulate diabetic corneal wound healing by promoting epithelial cell proliferation, migration and differentiation^1,17, 18^. Additionally, tear SP helps diabetic corneas recover corneal sensitivity by modulating neurogenic inflammation^17,18^. A decrease in tear SP levels has been found to be associated with an increase in the severity of DPN^18^. Similarly, tear NGF promotes corneal wound healing by decreasing neuroinflammation and stimulating epithelial cell proliferation and migration^1,19^. In our study, the tear SP and NGF concentrations were significantly lower in diabetic mice, but the levels increased significantly after 12 weeks of topical fenofibrate treatment. Oral fenofibrate did not alter tear SP or NGF levels, possibly because the therapeutic concentration achieved in the cornea was sub-optimal.

In diabetic corneas, corneal denervation reduces the blink reflex and decreases the viability of the epithelial cells due to diminished trophic support. On IVCM, we found diabetic mice had more variability in corneal epithelial cell size, wider intracellular space, more haze, and a lower CECD at baseline. This could be explained by the structural abnormalities attributed to the accumulation of advanced glycation end products (AGE). AGE buildup in diabetics decreases the adhesion in the corneal epithelium, resulting in morphological changes^20,21^. Another reason could be the loss of trophic support to epithelial cells as a result of diabetes-induced corneal denervation, potentially affecting corneal epithelial homeostasis^1^. After fenofibrate treatment, the morphology of epithelial cells improved in both the TF and OF groups, as evidenced by the restoration of regular cellular polygonal shape, decreased cell size, and increased CECD, all of which could be attributed to improvements in corneal nerve metrics and the restoration of trophic support to corneal epithelial cells.

Our results demonstrated that although diabetic mice presented significantly worse corneal epitheliopathy at baseline, it was reduced with either topical or oral fenofibrate treatment. Contrarily, mice in the PC group tended to develop neurotrophic keratopathy, which manifested as impaired corneal surface healing and focal corneal ulceration. Even though the reduction in corneal epitheliopathy was comparable after 12 weeks of topical and oral fenofibrate treatment, instilling fenofibrate eye drops effectively alleviated keratopathy earlier than oral fenofibrate treatment. It could be because the drug concentration on the targeted tissue, in this case, the cornea, is expected to be higher with topical fenofibrate instillation. It could also be due to the lubricant effect of fenofibrate eye drops, which accelerated ocular surface healing. Previous animal studies have also shown the promising efficacy of the topical application of PPAR agonists in corneal epithelial healing in an alkali-burn injury model, by inhibiting the inflammation and apoptosis of corneal epithelial cells^22–26^. Similarly, the GSEA showed that topical and oral fenofibrate treatment significantly reduced neutrophil formation, inhibiting inflammation-induced apoptosis of corneal epithelial cells while promoting their proliferation. Furthermore, fenofibrate eye drops upregulated the signalling pathways that involved stem cell pluripotency. Both alterations enhance corneal epithelial healing. Taken together, the improvement in corneal nerve metrics, restoration of trophic support, neuroactive interaction with corneal epithelial cells, and upregulation of anti-inflammatory and stem cell pluripotency pathways after fenofibrate treatment, ameliorated diabetic keratopathy.

CDC42 activation has been linked to neuronal apoptosis^27^, and its downregulation following fenofibrate treatment, whether topical or oral, suggests that fenofibrate has protective effects on neuronal survival. Hobson et al. also reported that decreasing CDC42 mRNA expression increased the neurite outgrowth from sensory neurons, supporting our findings^28^. β-III tubulin is an important component linked to axonal regeneration^29^. We found that topical fenofibrate treatment increased β-III-tubulin mRNA expression, leading to a significant difference in the β-III-tubulin levels between the TF and PC groups at 12 weeks. β-III tubulin-staining on the whole-mount diabetic corneas were also significantly increased after either topical or oral fenofibrate treatment.

The neuroprotective effects of fenofibrate on corneal nerves, whether topical or oral, are the result of several neurobiological pathways interacting. Based on the GSEA data, both topical and oral fenofibrate inhibited neutrophil formation and enhanced neuroactive ligand-receptor interaction. Consequently, the neuroinflammatory reactions ameliorated, and the corneal nerve status improved. Additionally, corneal necroptosis and apoptosis were significantly reduced after oral fenofibrate treatment. When topical fenofibrate treatment was compared to oral treatment, we found that the former resulted in significantly higher sphingolipid metabolism. Sphingolipids are highly enriched in the nervous system^30^, hence, the difference in the sphingolipid metabolism could be attributed to the improved corneal nerve parameters, including CNFL, CNFD, and TC after topical treatment, instead of just CNFD after oral treatment, thus increased nerve metabolic activity in the TF group. Lastly, diabetic mice had significantly lower cholesterol metabolism than non-diabetic mice (Supplementary Table 3). Previous studies have shown that DM impairs cholesterol metabolism^31,32^, accumulating long-chain fatty acids in nerve cell membranes that interfere with their function^33^, implying that diabetic patients are more likely to develop DPN. Tavakoli et al. also demonstrated the pathophysiological link between elevated triglyceride levels and corneal nerve damage^34^, suggesting the importance of regulating cholesterol metabolism to improve DCN prognosis.

Diabetic mice in our study showed significantly higher CCT due to hyperglycemia. DM causes an accumulation of AGEs in the cornea, resulting in structural abnormalities such as the formation of cytoplasmic vacuoles and corneal stromal edema, all of which may result in increased corneal thickness^35,36^. Hyperglycemia may also induce corneal endothelial dysfunction, resulting in stromal hydration^37^. Of note, it is expected that the CCT would not decrease after topical or oral fenofibrate treatment as we did not treat hyperglycemia throughout the study period. In addition, the PC group did not present a significant increase in CCT at week 12. We postulate that it might be partially due to the fact that 8 eyes in the PC group had central corneal ulceration.

The present study has several limitations. We instilled fenofibrate eye drops three times daily, as per previous publications on using fenofibrate eye drops to ameliorate ocular surface inflammation^22,24^. Detailed pharmacokinetic and pharmacodynamic studies on ocular tissue, as well as the measurements of drug concentrations in the corneas, to elucidate optimal dosing, will be conducted in the future. We will also study the relationship between the drug concentrations in corneas and its efficacy as well as adverse effects. Future studies could include comparative arms, such as treatment with topical recombinant human nerve growth factor (rhNGF), to compare its efficacy to topical and oral fenofibrate in ameliorating DCN. While topical fenofibrate is thought to be beneficial in corneas with down-regulated PPARα expression, for example, diabetic corneas, it is unclear whether the stimulating effects will benefit patients with normal PPARα expression in whom the corneal neuronal impairment is caused by an other etiology, such as corneal surgery. This would warrant further studies.

In conclusion, we demonstrated for the first time that fenofibrate eye drops had neurotrophic effects, while oral fenofibrate also provided favorable effects on DCN. Compared to oral fenofibrate, fenofibrate eye drops showed earlier beneficial effects on diabetic keratopathy and reduced neuroinflammation. Our findings highlight the therapeutic potential of topical and oral fenofibrate in the treatment of DCN.

## Methods

### Experimental groups

Thirty wild-type and 90 Ins2Akita male mice (Jackson Laboratory, Bar Harbor, US) were used. Their blood glucose levels were measured monthly using a glucometer. Mice were divided into four groups (n = 60 eyes for each): (1) negative control (wild-type mice), (2) positive control (PC) (Akita mice), (3) topical fenofibrate (TF) group : akita mice received 0.05% fenofibrate hydrochloride ophthalmic solution, three times daily, (4) oral fenofibrate (OF) group: akita mice received fenofibrate via oral gavage (50 mg/kg) once daily^6,14^. The fenofibrate eye drops were prepared using 0.1 mL polyoxyethylene sorbitan monooleate and 100 mL NaCl-based phosphate-buffered saline (PBS), and then 10 mg of fenofibrate was dissolved in 20 mL of vehicle solution to formulate the 0.05% fenofibrate eye drops^23^. Whereas, the oral gavage was prepared by dissolving fenofibrate in 0.05% carboxymethylcellulose at 2 mg/1 mL, i.e. 0.05%. Subsequently, each mouse was fed 0.1 mL of fenofibrate gavage^14^. All animals used were treated in accordance with tenets of the Association for Research in Vision and Ophthalmology Statement, and the Institutional Animal Care and Use Committee of SingHealth approved the protocol (2020/SHS/1612). Half of the mice from each group were euthanized at 6 weeks, and the other half were euthanized at 12 weeks, with an overdose of intraperitoneal pentobarbitone (60–150 mg/kg) administered under general anesthesia. Baseline was defined as the parameters evaluated at the start of the study, i.e. before the fenofibrate treatment. The authors confirm that the study is reported in accordance with the ARRIVE (Animal Research: Reporting of In Vivo Experiments) guidelines.

### Corneal sensitivity

The corneal sensitivity was measured using a Cochet and Bonnet Aesthesiometer. The nylon filament was extended to its maximum length (6.0 cm) and shortened by 0.5 cm. The filament length that prompted an eye-blink response was recorded. Five different areas, including the central, superior, inferior, nasal and temporal parts of the cornea, were evaluated (0–30 cm).

### In vivo confocal microscopy (IVCM)

Each cornea was scanned in five areas: central, superior, inferior, nasal and temporal parts of the cornea. Five best-focused images of subbasal nerves were selected for each area, and these 25 micrographs selected for each eye were analyzed ^38,39^ using CCMetrics software (University of Manchester, Manchester, UK)^40^. The following parameters were obtained: CNFD, computed as the number of fibres/mm^2^; corneal nerve branch density (CNBD), computed as the number of branch points on the main fibres/mm^2^; corneal nerve fibre length (CNFL), calculated as the total length of fibres (mm)/mm^2^. The tortuosity of nerve fibres was computed as the tortuosity coefficient (TC). Five best-focused micrographs with clearly visible cell borders from each eye were selected for corneal epithelium analysis. The cell density (cells/mm^2^) was manually calculated by using ImageJ.

### Slit lamp assessment of ocular surface

Topical minims fluorescein sodium 2.0% was instilled to visualize the devitalized areas of the corneal surface using a slit lamp biomicroscope. Corneal epitheliopathy was quantified based on the percentage (%) of fluorescein-positive surface area (F+) as a function of total corneal area (T) (corneal epitheliopathy% = F+/T) using ImageJ.

### Anterior segment optical coherence tomography (ASOCT) evaluation

Three high-resolution corneal scans of ASOCT examination (Optovue, Inc., Fremont, USA) were obtained for each eye at each time point to evaluate the central corneal thickness (CCT) using an in-built scale^42^, as well as the pathological corneal changes.

### Immunohistochemistry (IHC) staining

The corneal sections were prepared as we previously described^43,44^. The sections were stained with primary antibody labelling of rabbit polyclonal anti-PPARα (1:200) (PA1-822A; Invitrogen; Waltham, USA) at 4 °C overnight. After three washes with 1× PBS, the sections were incubated with goat anti-rabbit AlexaFluor 488-conjugated secondary antibody (Life Technologies, Carlsbad, USA) in 1× PBS-BSA at room temperature. Sections were then mounted with DAPI containing UltraCruiz Mounting Medium.

### Whole mount IHC

The corneas were fixed in 4% paraformaldehyde, followed by washes with 1xPBS. The corneas were quenched before non-specific binding incubation in 10% normal goat serum plus 0.3% Triton X-100 in 0.1% 1× PBS-BSA. Then corneas were doubled stained with primary antibodies rabbit polyclonal anti-β-III tubulin (1:1000) (BioLegend Inc., SanDiego, USA) for 72 h^45^. The corneas were then incubated with the corresponding secondary antibodies in 1xPBS-BSA for 24 h and then washed before mounting with UltraCruz mounting medium containing DAPI. Positive staining of β-tubulin was quantified based on the percentage of positively stained surfaced area to the area of the whole corneal area that was stained with DAPI. The quantification was carried out using ImageJ.

### Western blot analysis

The excised corneas were lysed and homogenized. 30 µg proteins from each sample were resolved on SDS-PAGE and transferred to a PVDF membrane. Then, the membranes were blocked with 5% non-fat milk in PBS containing 0.1% Tween 20 and then probed with primary antibodies (rabbit polyclonal PPARα; 1:500) in 1% non-fat milk containing PBS overnight at 4 °C. The membranes were then washed with 0.1% PBS and incubated with goat anti-rabbit horseradish peroxidase-conjugated secondary antibody (1: 3000; Cell Signaling Technology) in 1% milk in PBS. Protein bands were visualized using SuperSignal TM West Pico PLUS chemiluminescent detection reagents and quantified using ImageJ.

### Trigeminal ganglion quantitative real-time polymerase chain reaction (PCR) analysis

Total RNA was isolated from fresh trigeminal ganglion^46^, where it was cut into small pieces and transferred into ice-cold TRIzol reagent, followed by homogenization. Total RNA was extracted using the PureLink Mini Kit (Ambion, Life Technologies) and was quantified using a NanoDrop ND-1000 Spectrophotometer. Total RNA (140 ng) from each sample was reverse transcribed into cDNA using Superscript III (Thermos Scientific), followed by qRT-PCR, which was carried out with a total volume of 10uL containing LightCycler 480 SYBR Green Master (Roche, Switzerland), primers, PCR Grade water and cDNA. The following primers were used: β-III-Tubulin (Tuj1), NGF, CDC42 and GAP43 (Supplementary Table 1). Threshold cycles (CT) were normalized to the expression of the housekeeping gene, Glyceraldehyde-3-Phosphate Dehydrogenase (GAPDH). Relative fold changes were analyzed by the ΔΔCT method.

### Tear enzyme-linked immunosorbent assay (ELISA) analysis

The tear collection and tear protein extraction were performed as previously described^47–49^. Briefly, tear proteins were extracted from the Schirmer’s strip sample by immersing cut Schirmer’s strips into an elution buffer that contained 0.33% Tween-20, 0.55 M NaCl, 0.55% BSA and protease inhibitor. The elution buffer underwent sonication and agitation at 450 rpm for 17 h at 4 °C. Subsequently, we centrifuged the elutes and collected the clear supernatants for protein assay with a Micro BCA Protein Assay Kit (Pierce Biotechnology, Inc. USA). ELISA for NGF, Substance P (SP) and Calcitonin gene-related peptide (CGRP) was then carried out, with the details provided in Supplementary Table 2^49^.

### Tear proteomic analysis

The sample preparation and peptide extraction were performed with published protocols^49,50^. Briefly, small pieces of the Schirmer’s strips were soaked in 100 μl lysis buffer, and the solution was mixed in a Thermomixer for 1.5 h at 20 °C. Subsequently, the Bio-Rad DC Protein assay measured the total protein concentration. Using the Thermo Easy Mini sample prep kits, 100 μg of tear proteins from each sample were reduced, alkylated, digested with trypsin, and later desalted. A Fluorometric peptide quantification kit measured the total peptide amount. A SpeedVac dried the extracted peptide sample that was later resuspended in 0.1%FA/2% acetonitrile (ACN) containing indexed Retention Time (iRT) (1:10). All the peptide samples were processed using an EASY-nLC 1200 system equipped with an Orbitrap Exploris™ 480 Mass Spectrometer. An Acclaim PepMap 100 C18 was used as a pre-column and a PepMap^®^RSLC C18 as an analytical column. The samples were separated on a capillary column against a gradient, where 0.1% formic acid (FA) was used as mobile phase A, and 80% ACN in 0.1% FA as mobile phase B. Data independent acquisition (DIA) experiments were then conducted. The results were then processed via library-free directDIA workflow in Spectronaut 15. Following the mapDIA quality control criteria^51^, fragment ions were isolated for quantification, and the raw protein abundance values were derived.

### Statistical analysis

All data were expressed as mean ± standard deviation. A linear mixed model was used to analyze the data at different time points from both eyes, as well as the data of four groups, to take into account the correlation of the time points and both eyes. All the statistical analyses were performed using STATA software (STATACrop, College Station, TX). The p-values < 0.05 were considered statistically significant.

We used the custom scripts in R (64-bit version 4.1.1) for the proteomic data to analyze the downstream data and perform Gene Ontology term enrichment. The raw protein abundance values were median-normalized and log-transformed. Subsequently, we performed the Gene Set Enrichment Analysis (GSEA) with the use of the KEGG database as a reference source.^50,52^ The enrichment score (ES) represents a standard Kolmogorov–Smirnov statistic, and shows the degree to which genes in a gene set are overexpressed (positive ES) or underexpressed (negative ES), reflecting positive or negative correlations, respectively.

### Supplementary Information

Supplementary Information.

Supplementary Figure 1.

Supplementary Figure 2.

## Data Availability

All data generated or analysed during this study are included in this manuscript and its supplementary information files.
