# Supplementary material for: Topical and oral peroxisome proliferator-activated receptor-α agonist ameliorates diabetic corneal neuropathy
Source: Sci Rep. 2024 Jun 11;14:13435. doi: 10.1038/s41598-024-64451-4 (PMC11167005; doi:10.1038/s41598-024-64451-4)
Supplement: Supplementary file 1 — Supplementary Information. [file 41598_2024_64451_MOESM1_ESM.pdf]

**Topical and Oral Peroxisome Proliferator-activated Receptor-α Agonist Ameliorates Diabetic Corneal Neuropathy**

Hassan Mansoor, Isabelle Xin Yu Lee, Molly Tzu-Yu Lin, Heng Pei Ang, Yao Cong Xue, L Krishaa, Moushmi Patil, Siew-Kwan Koh, Hong Chang Tan, Lei Zhou,
Yu-Chi Liu.

**Supplementary Figure Legends:**

**Supplementary Figure 1: Blood glucose levels of mice in different experimental groups.** Diabetic mice, including PC, TF and OF groups, presented significantly higher blood glucose levels than the NC group at different time points. *** represents $p<0.001$. *** represents statistical significance for intergroup comparisons.

**Supplementary Figure 2: Scatter plots for GSEA results.** The GSEA plots (ES and -log10 [p value]) showing the enrichment pathways ($p<0.05$) comparing **(A)** PC versus NC groups at baseline **(B)** TF versus OF groups at 12 weeks**.** Red dots represent the significantly up-regulated pathways, and blue dots represent significantly down-regulated pathway.

(a)

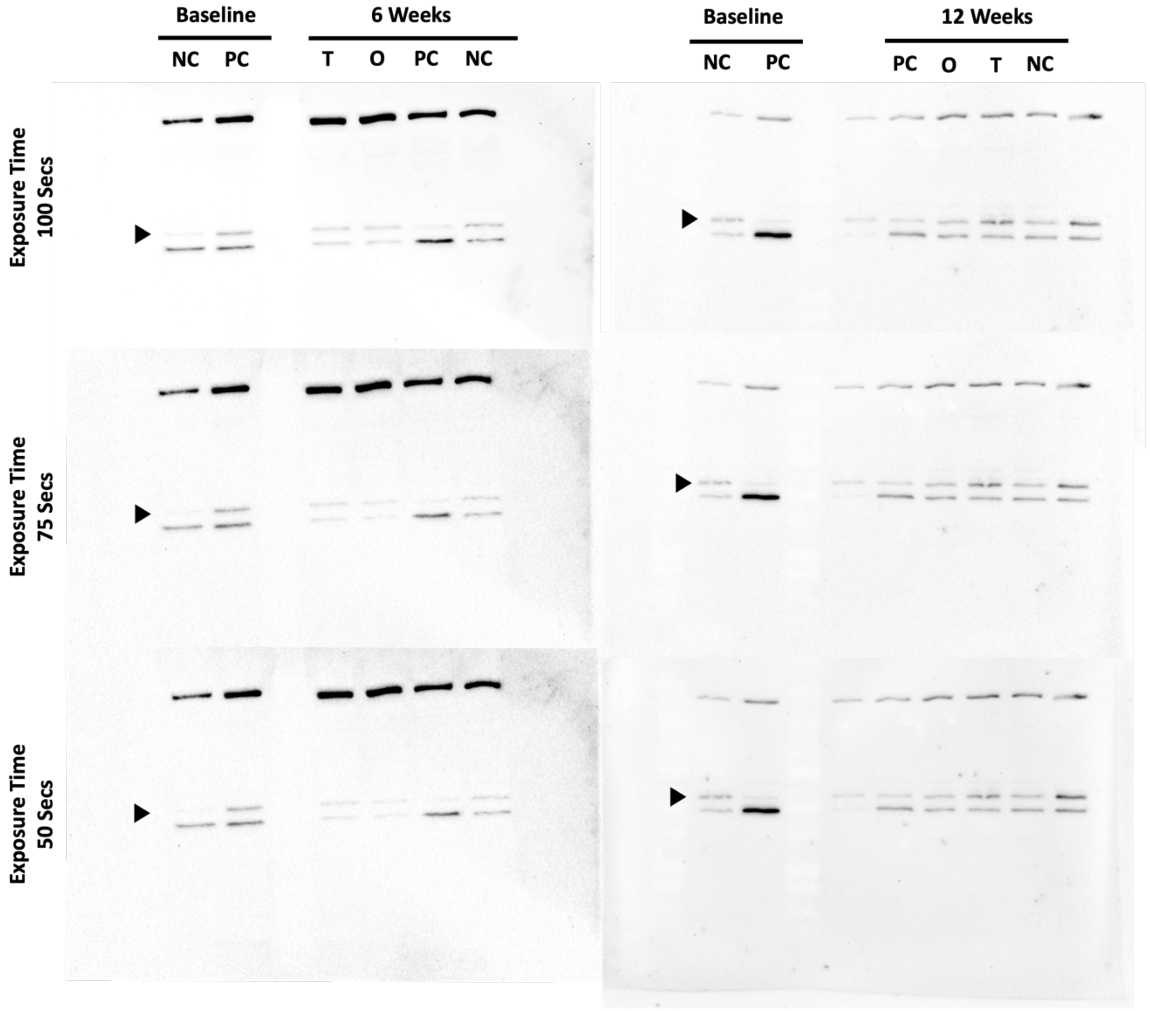
Baseline
6 Weeks
NC PC
T O PC NC
Baseline
12 Weeks
NC PC
PC O T NC
Exposure Time 100 Secs
Exposure Time 75 Secs
Exposure Time 50 Secs

(b)

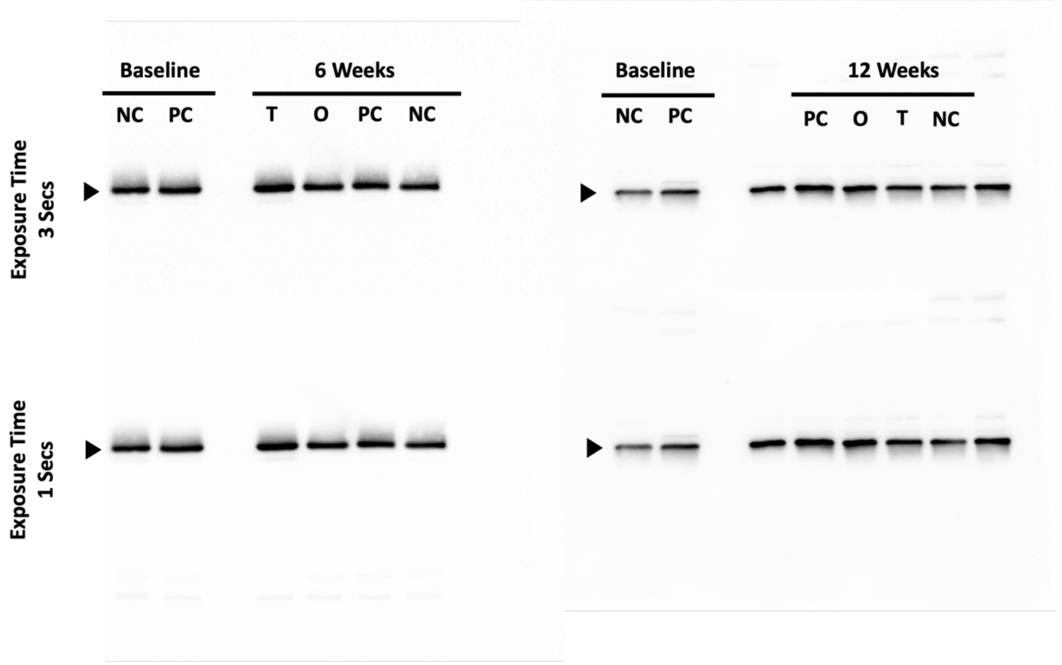
Baseline
6 Weeks
NC PC
T O PC NC
Baseline
12 Weeks
NC PC
PC O T NC
Exposure Time 3 Secs
Exposure Time 1 Secs

**Supplementary Figure, S1; Uncropped image of Western Blot for the expression of PPARα present in wild-type mice (negative control), diabetic mice (positive control), diabetic mice with topical fenofibrate (Topical treatment) and diabetic mice with oral fenofibrate (Oral treatment).** (a) The expression of PPARα at 58kD with different exposure times. (b) The expression of β-actin (42kDa) with different exposure times. Purified protein was evaluated running on SDS-PAGE gels. PVDF membranes were cut prior to hybridization with rabbit polyclonal PPARα (1:500, 58kDa) overnight at 4˚C before further incubation with goat anti-rabbit horseradish peroxidase-conjugate secondary antibody (1:3000). The molecular weight standard use was precision plus protein dual color standard (Cat. No.: #1610374; Bio-Rad). To ensure that the same amount of protein was added, β-actin (42kDa) was introduced to normalize the level of protein detected. Original whole western blot is shown for baseline, week 6 and week 12 results for each group. Exposure time used in the manuscript: 100 secs for PPARa bands, and 1 sec for b-actin bands.

**Supplementary Tables**

**Supplementary Table 1: Primer used in trigeminal ganglion quantitative real-time polymerase chain reaction (PCR) analysis.**

| Primer | Forward Sequence | Reverse Sequence |
|---|---|---|
| β-III-Tubulin (Tuj1) | CGCCTGCCTTTTCGTCTCTA | CTCATCGCTGATGACCTCCC |
| NGF | GTAGACCCCAGACTGTTTAAGAAACG | GTACCATGGGCCTGGAAGTCTAG |
| CDC 42 | GAGTGCTCTGCCCTCACAC | GGCTCTTCTTCGGTTCTGG |
| GAP 43 | GGAGAAGGATGATGCTCCCG | TTTGGCTTCGTCTACAGCGT |
| GAPDH (housekeeping gene) | AGGTCGGTGTGAACGGATTTG | TGTAGACCATGTAGTTGAGGTCA |

**Supplementary Table 2: Information of ELISA Kits & Dilution Factors for Tears.**

| Neuromediators | | Kit and dilution factor |
|---|---|---|
| 1 | Substance P (SP) | 6x dilution; R&D Systems, Minneapolis, USA |
| 2 | Beta-Nerve Growth Factor (β-NGF) | 2.5x dilution; RayBiotech Life, Inc., GA, USA |
| 3 | Calcitonin Gene Related Peptide (CGRP) | 3x dilution; Phoenix Pharmaceuticals, Runcorn, UK |

**Supplementary Table 3: Top 10 significantly expressed pathways involved by significantly up-regulated and down-regulated tear proteins in the PC group in comparison to those in the NC group.**

| Pathway Name | | Enrichment Score | P Value |
|---|---|---|---|
| 1 | Complement and coagulation cascades | -0.7787 | 0.002 |
| 2 | Cholesterol metabolism | -0.7615 | 0.004 |
| 3 | Notch signalling pathway | 0.9922 | 0.006 |
| 4 | Adipocytokine signalling pathway | 0.9711 | 0.006 |
| 5 | Type I diabetes mellitus | 0.9934 | 0.006 |
| 6 | Non-alcoholic fatty liver disease | 0.5900 | 0.009 |
| 7 | Lysosome | -0.5601 | 0.016 |
| 8 | Longevity regulating pathway | 0.8178 | 0.016 |
| 9 | Purine metabolism | 0.5281 | 0.020 |
| 10 | Circadian rhythm | 0.9100 | 0.022 |

**Supplementary Table 4: Top 10 significantly expressed pathways involved by significantly up-regulated and down-regulated tear proteins in the TF group after 12-weeks of treatment versus baseline.**

| | Pathway Name | Enrichment Score | P value |
|---|---|---|---|
| 1 | Folate biosynthesis | 0.8326 | 0.006 |
| 2 | Vitamin digestion and absorption | 0.8268 | 0.010 |
| 3 | IL-17 signalling pathway | -0.7423 | 0.011 |
| 4 | Drug metabolism - cytochrome P450 | 0.5066 | 0.015 |
| 5 | Neutrophil extracellular trap formation | -0.6111 | 0.015 |
| 6 | Signalling pathways regulating pluripotency of stem cells | 0.7504 | 0.016 |
| 7 | Neuroactive ligand-receptor interaction (Neurofunction) | 0.8234 | 0.019 |
| 8 | Natural killer cell-mediated cytotoxicity | -0.7725 | 0.029 |
| 9 | Diabetic cardiomyopathy | -0.5222 | 0.031 |
| 10 | Renin secretion | 0.6983 | 0.040 |

**Supplementary Table 5: Top 10 significantly expressed pathways involved by significantly up-regulated and down-regulated tear proteins in the OF group after 12-weeks of treatment versus baseline.**

| Pathway Name | | Enrichment Score | P Value |
|---|---|---|---|
| 1 | Neutrophil extracellular trap formation | -0.6021 | 0.004 |
| 2 | Ubiquitin mediated proteolysis | -0.6954 | 0.006 |
| 3 | Neuroactive ligand-receptor interaction | 0.7985 | 0.014 |
| 4 | Starch and sucrose metabolism | -0.6980 | 0.023 |
| 5 | Intestinal immune network for IgA production | 0.9404 | 0.029 |
| 6 | Apoptosis | -0.8835 | 0.039 |
| 7 | Endocrine and other factor-regulated calcium reabsorption | 0.6244 | 0.041 |
| 8 | Protein digestion and absorption | -0.7983 | 0.045 |
| 9 | Necroptosis | -0.5095 | 0.047 |
| 10 | Renin-angiotensin system | 0.6219 | 0.047 |

**Supplementary Table 6: Significantly expressed pathways involved by significantly up-regulated and down-regulated tear proteins in the TF group versus OF group after 12 weeks of treatment.**

| | Pathway Name | Enrichment Score | P Value |
|---|---|---|---|
| 1 | Intestinal immune network for IgA production | 0.9694 | 0.006 |
| 2 | Sphingolipid metabolism | 0.8605 | 0.008 |
| 3 | Carbohydrate digestion and absorption | -0.8371 | 0.021 |
| 4 | Lysosome | 0.5551 | 0.029 |
| 5 | Longevity regulating pathway | -0.8231 | 0.029 |
| 6 | Adipocytokine signalling pathway | -0.9366 | 0.043 |
| 7 | Fc gamma R-mediated phagocytosis | -0.5664 | 0.045 |
