# Supplementary figures and images for: Topical and oral peroxisome proliferator-activated receptor-α agonist ameliorates diabetic corneal neuropathy
Source: Sci Rep. 2024 Jun 11;14:13435. doi: 10.1038/s41598-024-64451-4 (PMC11167005; doi:10.1038/s41598-024-64451-4)

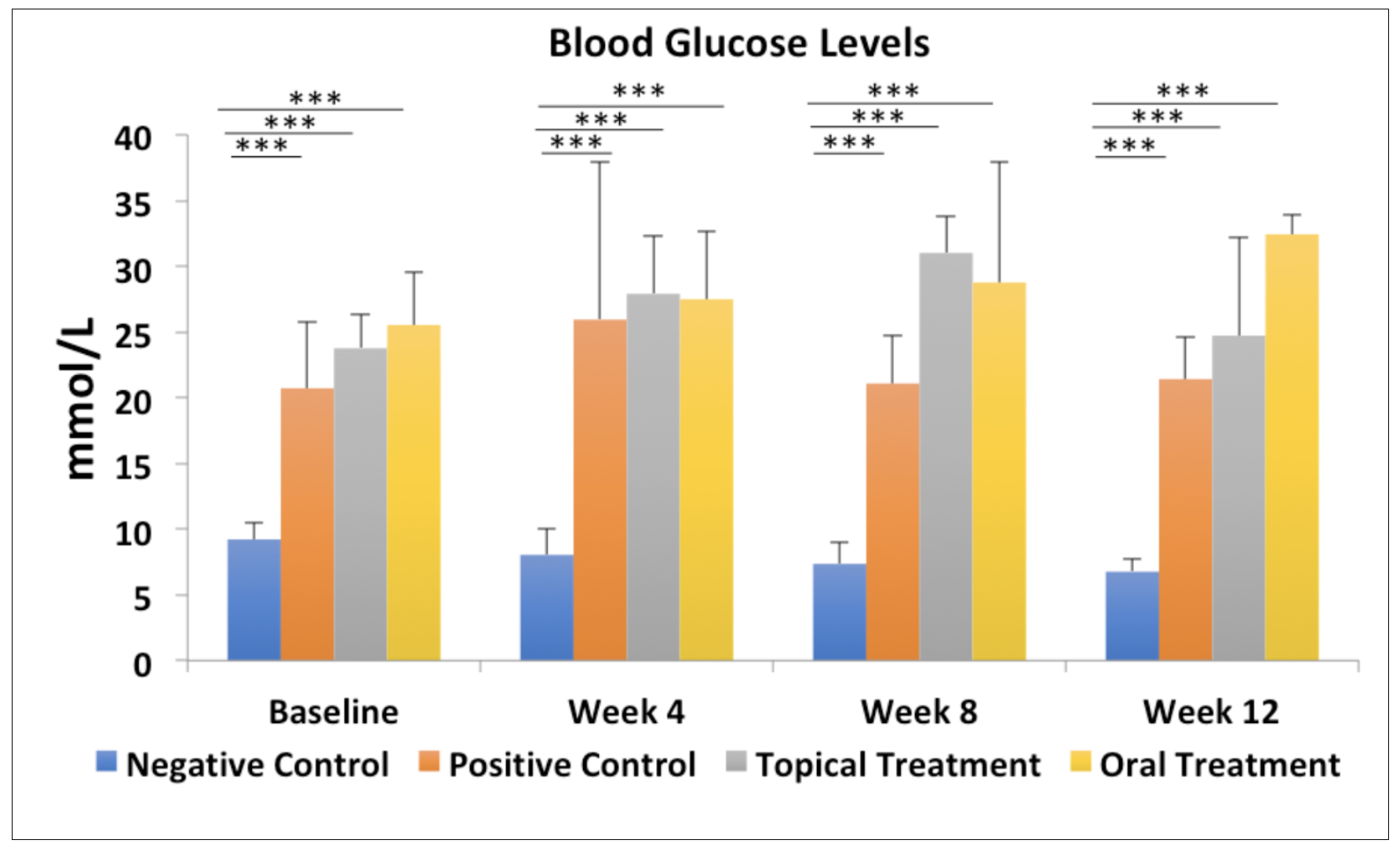

Supplement: Supplementary file 2 — Supplementary Figure 1. [file 41598_2024_64451_MOESM2_ESM.tiff]

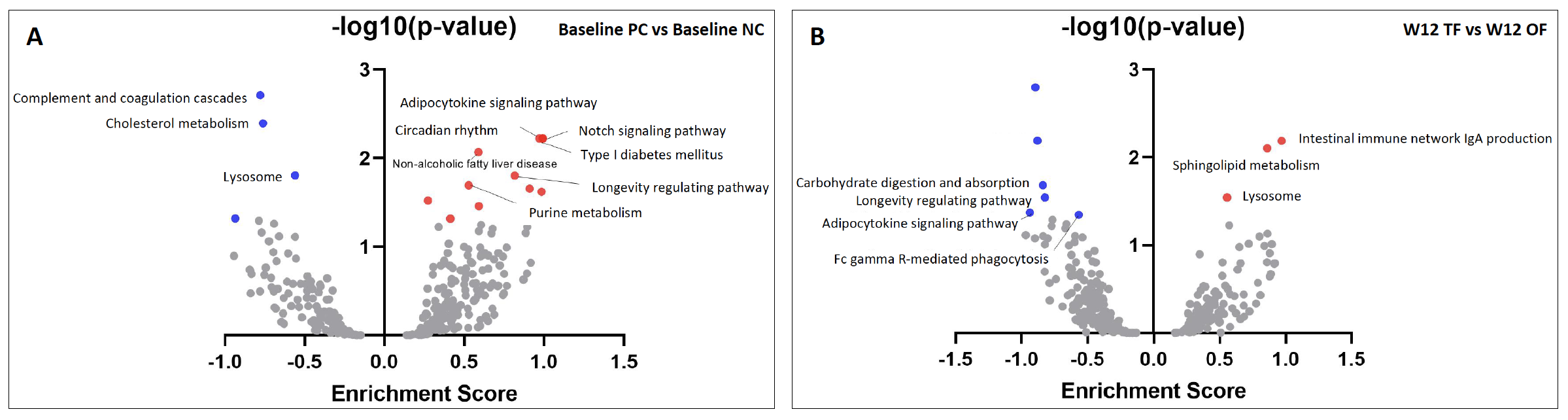

Supplement: Supplementary file 3 — Supplementary Figure 2. [file 41598_2024_64451_MOESM3_ESM.tiff]
